# Nebulized Epinephrine Versus Placebo in Infants with Moderate-to-Severe Bronchiolitis: A Randomized Controlled Trial

**DOI:** 10.3390/children13091254

**Published:** 2026-09-16

**Authors:** Immacolata Rulli, Simone Foti Randazzese, Fabio Toscano, Lucia Marina Marseglia, Sara Manti, Eloisa Gitto

**Affiliations:** 1 Neonatal and Pediatric Intensive Care Unit, “Gaetano Martino” Hospital, Via Consolare Valeria 1, 98124 Messina, Italy; immacolata.rulli@polime.it; 2 Pediatric Unit, Department of Human Pathology of Adult and Childhood “Gaetano Barresi”, University of Messina, Via Consolare Valeria 1, 98124 Messina, Italy; toscanoftp@gmail.com; 3 Neonatal and Pediatric Intensive Care Unit, Department of Clinical and Experimental Medicine, University of Messina, Via Consolare Valeria 1, 98124 Messina, Italy; luciamarina.marseglia@unime.it (L.M.M.); egitto@unime.it (E.G.)

**Keywords:** bronchiolitis, children, nebulized epinephrine, pediatric intensive care unit

## Abstract

**Highlights:**

**What are the main findings?**

- In infants with moderate-to-severe bronchiolitis requiring Pediatric Intensive Care Unit admission, nebulized epinephrine did not demonstrate superiority over placebo in improving oxygenation or reducing respiratory and heart rates.
- Clinical improvement over time occurred in both groups and was likely attributable to optimized supportive care.

**What are the implications of the main findings?**

- Routine administration of nebulized epinephrine in severe bronchiolitis requiring intensive respiratory support is not supported by our findings.
- Management strategies should prioritize evidence-based supportive care, while future larger multicenter trials are needed to identify potential subgroups that may benefit from targeted pharmacologic intervention.

**Abstract:**

**Background:** International guidelines do not support the routine use of nebulized epinephrine for acute viral bronchiolitis. Despite its vasoconstrictive and bronchodilator properties, evidence remains inconclusive. **Objectives:** Day-6 fraction of inspired oxygen (FiO_2_) adjusted for baseline FiO_2_ was designated as the principal outcome; however, this outcome hierarchy was not prospectively prespecified. The analysis compared day-6 FiO_2_, adjusted for baseline FiO_2_, between nebulized racemic epinephrine and placebo; peripheral capillary oxygen saturation (SpO_2_), respiratory rate (RR), and heart rate (HR) were secondary outcomes. **Methods:** This randomized, single-blind, placebo-controlled trial included 26 infants aged 0–12 months with moderate-to-severe bronchiolitis requiring respiratory support. Participants were allocated 1:1 to nebulized racemic epinephrine (group A, *n* = 13) or isotonic saline placebo (group B, *n* = 13). Outcomes were measured at baseline (T0), day 3 (T1), and day 6 (T2). The primary analysis used ANCOVA to compare T2 FiO_2_ while adjusting for T0 FiO_2_; mixed repeated-measures ANOVA assessed longitudinal trajectories. **Results:** The adjusted T2 FiO_2_ difference between groups was −0.43 percentage points (95% CI −3.43 to 2.57; *p* = 0.772). Adjusted T2 differences were also non-significant for SpO_2_ (*p* = 0.058), RR (*p* = 0.093), and HR (*p* = 0.487). FiO_2_ decreased over time (*p* = 1.99 × 10^−4^), but the group-by-time interaction was not significant (*p* = 0.108); no interaction was observed for SpO_2_, RR, or HR (*p* = 0.296, *p* = 0.187, and *p* = 0.352, respectively). **Conclusions:** Nebulized racemic epinephrine did not improve the trajectories of oxygen requirement, SpO_2_, RR, or HR compared with placebo; improvements over time were observed in both groups.

## 1. Introduction

Acute viral bronchiolitis represents the most common lower respiratory tract infection in newborns and infants and remains the leading cause of hospitalization in this age group worldwide [1]. It is characterized by inflammation of the small airways, epithelial cell necrosis, mucus hypersecretion, and airway edema, ultimately resulting in airflow obstruction and impaired gas exchange [2].

The main etiological agent is the Respiratory Syncytial Virus (RSV), a single-stranded negative-sense RNA virus belonging to the *Pneumoviridae* family, *Orthopneumovirus* genus [3]. In addition to RSV, isolated infections or co-infections with other viruses, including Orthomyxovirus, Severe Acute Respiratory Syndrome Coronavirus 2 (SARS-CoV-2), Rhinovirus, Metapneumovirus, and Adenovirus, as well as bacterial pathogens such as *Haemophilus influenzae* and *Streptococcus pneumoniae*, have also been reported [4,5]. RSV is responsible for approximately 40% of hospitalizations for pneumonia in infants and 50% of cases of bronchiolitis in children under 5 years of age, of whom 80% are younger than 1 year. The highest mortality rates are observed in low- and middle-income countries, accounting for over 95% of affected pediatric cases [6]. Among infants aged 0–6 months, RSV infection is estimated to cause about 6.6 million episodes of bronchiolitis, 1.4 million hospitalizations, 13,300 in-hospital deaths, and 45,700 deaths outside hospital settings annually [6].

The substantial epidemiological impact of RSV is primarily attributed to its high basic reproduction number (R_0_), estimated at 4.5, which exceeds that of influenza virus (R_0_ ≈ 1.2) and the SARS-CoV-2 wild-type variant (R_0_ ≈ 2.5), and is second only to that of measles virus (R_0_ ≈ 18) [7].

Several studies have also demonstrated that 30–40% of children hospitalized for RSV bronchiolitis are predisposed to developing medium- and long-term respiratory complications, such as recurrent wheezing, impaired pulmonary function persisting into adolescence, and increased risk of asthma [8,9,10,11].

Current international guidelines recommend primarily supportive management of bronchiolitis, including oxygen supplementation, hydration, and nutritional support, while discouraging the routine use of pharmacological interventions such as bronchodilators, corticosteroids, or nebulized epinephrine. However, the pathophysiological features of bronchiolitis—namely airway edema, increased mucus production, and variable bronchial smooth muscle constriction—have prompted investigation into therapies targeting these mechanisms [1,12,13].

Epinephrine, an endogenous catecholamine, exerts its pharmacological effects through stimulation of α- and β-adrenergic receptors [14]. Activation of α_1_-adrenergic receptors induces vasoconstriction of the bronchial mucosal vasculature, potentially reducing airway edema and improving luminal patency [15]. Concurrent stimulation of β_2_-adrenergic receptors promotes bronchodilation via relaxation of airway smooth muscle and may enhance mucociliary clearance [16]. Additionally, β-adrenergic signaling has been suggested to modulate inflammatory mediator release, although this effect remains incompletely characterized in the context of viral bronchiolitis [17].

Despite these theoretically beneficial mechanisms, clinical evidence regarding the efficacy of nebulized epinephrine in bronchiolitis remains inconsistent. Consequently, its use remains controversial, particularly in moderate-to-severe cases requiring respiratory support.

To address this uncertainty, we conducted a randomized, single-blind, placebo-controlled trial. The primary efficacy outcome was defined as fraction of inspired oxygen (FiO_2_) at day 6 adjusted for baseline FiO_2_; however, this outcome hierarchy was not prospectively prespecified. Peripheral capillary oxygen saturation (SpO_2_), respiratory rate (RR), and heart rate (HR) at day 6, adjusted for their corresponding baseline values, were secondary outcomes. Longitudinal trajectories from T0 to T2 and the duration of respiratory support and hospitalization were exploratory outcomes.

## 2. Subjects and Methods

### 2.1. Study Design and Setting

This randomized, single-blind, placebo-controlled clinical study was conducted between February and April 2025 in the Pediatric Intensive Care Unit (PICU) of the “Gaetano Martino” University Hospital, Messina, Italy.

### 2.2. Study Population

Infants aged 0–12 months and receiving a diagnosis of moderate-to-severe bronchiolitis were recruited. Diagnosis and severity stratification were performed in accordance with the updated 2022 Italian intersociety guidelines for bronchiolitis management [1]. Consistent with these recommendations, bronchiolitis was diagnosed clinically based on history and physical examination. Disease severity was defined according to the degree of respiratory compromise, oxygen requirement, and feeding impairment, with particular attention to persistent oxygen saturation <92%, increased work of breathing, and inability to maintain adequate oral hydration [1].

Exclusion criteria included the presence of significant comorbidities that could confound respiratory outcomes, such as congenital heart disease, cystic fibrosis, primary ciliary dyskinesia, chromosomal abnormalities, inherited metabolic disorders, and known primary or secondary immunodeficiencies.

### 2.3. Randomization and Blinding

All participants were randomly assigned in a 1:1 ratio to receive either nebulized racemic epinephrine (group A) or a placebo (group B), alongside feeding and respiratory support.

Randomization was performed using a block randomization method to ensure balanced group allocation.

The study followed a single-blind design: the principal investigator responsible for clinical assessment and data analysis remained blinded to group allocation throughout the study period, while treatment administration was performed by non-blinded clinical staff.

### 2.4. Intervention

Randomization concerned the nebulized study treatment only. Both groups otherwise received supportive care as clinically indicated, including nutritional support and respiratory assistance.

Patients in group A received nebulized racemic epinephrine (0.02 mL/kg of a 1% solution diluted to 6 mL with normal saline) every 6 h [18], whereas group B received an equivalent volume of nebulized normal saline on the same schedule. The assigned treatment was continued through day 6 and was therefore ongoing at both follow-up assessments: FiO_2_, SpO_2_, RR, and HR were measured at baseline (T0), on day 3 during treatment (T1), and on day 6 during treatment (T2).

### 2.5. Data Collection and Clinical Variables

Baseline demographic and clinical characteristics were collected for all participants, including sex, ethnicity, gestational age at birth, and breastfeeding status. Additional clinical data comprised the etiological agent(s) associated with bronchiolitis, when available, as well as disease severity assessed using a validated bronchiolitis severity score (BSS) [19], length of hospital stay, and the type and duration of respiratory support required.

FiO_2_ at T2, adjusted for T0 FiO_2_, was selected as the primary efficacy outcome because it directly quantifies the oxygen concentration required to maintain adequate oxygenation while accounting for baseline oxygen requirement. This outcome hierarchy was not prospectively prespecified. SpO_2_, RR, and HR at T2, adjusted for their corresponding T0 values, were secondary outcomes because together they capture oxygenation, respiratory distress, and cardiorespiratory response. Longitudinal T0–T2 trajectories, length of hospitalization, duration and type of respiratory support, and adverse events were analyzed as exploratory outcomes.

### 2.6. Statistical Analysis

The data were analyzed using Microsoft Excel (version 2023) and the Statistical Package for the Social Sciences software (version 22.0). Continuous variables are reported as mean (SD); because age was markedly skewed, it is additionally summarized as median (interquartile range). Categorical variables are reported as *n* (%). In accordance with Consolidated Standards of Reporting Trials (CONSORT) reporting principles, baseline characteristics were described using absolute standardized differences rather than significance tests. The primary analysis compared T2 FiO_2_ between randomized groups using analysis of covariance (ANCOVA), with treatment group as the independent variable and T0 FiO_2_ as a covariate. The same baseline-adjusted ANCOVA approach was applied to the secondary T2 outcomes (SpO_2_, RR, and HR). Adjusted mean differences with 95% confidence intervals are reported. A Welch comparison of T0-to-T2 change scores was used as a sensitivity analysis for the primary outcome. Additional exploratory FiO_2_ sensitivity models separately adjusted for log-transformed age or exclusive breastfeeding; both covariates were not entered simultaneously because of the small sample. Longitudinal trajectories were assessed using mixed repeated-measures analysis of variance (ANOVA) with treatment group as the between-subject factor, time (T0, T1, T2) as the within-subject factor, and the group-by-time interaction; Greenhouse–Geisser correction was applied to time and interaction tests. Exploratory between-group contrasts at each time point used Welch’s *t*-tests with Holm adjustment within each outcome. Hospitalization and respiratory-support durations were compared exploratorily using Welch’s *t*-tests with mean differences and 95% confidence intervals; Mann–Whitney tests were used as distribution-robust sensitivity analyses. All tests were two-sided, and *p* < 0.05 was considered statistically significant. Exact numerical *p*-values are reported. No formal a priori sample-size calculation was performed, and the endpoint hierarchy and analysis plan were not prespecified; all inferential analyses should therefore be interpreted as exploratory.

### 2.7. Ethical Considerations

The study was designed and conducted in compliance with Good Clinical Practice standards and adhered to the ethical principles outlined in the Declaration of Helsinki and its subsequent amendments. Ethical committee approval was obtained (num. 4-23, 24 July 2023). Informed consent was obtained from the caregivers of all patients involved in the study, in accordance with the study protocol. The trial was not prospectively registered in a public trial registry.

## 3. Results

All 26 randomized participants were included in the analysis (Figure S1): 13 received nebulized racemic epinephrine (group A) and 13 received isotonic saline placebo (group B). Complete T0, T1, and T2 data were available for all four physiological outcomes.

Baseline characteristics and exploratory post-randomization outcomes are summarized in separate panels of Table 1. Potentially important chance baseline imbalances were present: age at PICU admission was lower in group A (median 37 [IQR 19–61] days) than in group B (68 [39–93] days; absolute standardized difference 0.50), and exclusive breastfeeding was less frequent in group A (3/13, 23.1%) than in group B (8/13, 61.5%; absolute standardized difference 0.85). These imbalances may have reduced precision; their potential influence on the primary result was examined in separate sensitivity analyses.

For the primary analysis, the adjusted mean difference in T2 FiO_2_ between group A and group B was −0.43 percentage points (95% CI −3.43 to 2.57; *p* = 0.772). A sensitivity analysis comparing T0-to-T2 change scores yielded a between-group difference of 0.77 percentage points (95% CI −4.77 to 6.31; *p* = 0.772). Separate sensitivity models additionally adjusting for log-transformed age (adjusted difference −0.56, 95% CI −3.71 to 2.58; *p* = 0.713) or exclusive breastfeeding (−0.16, 95% CI −3.50 to 3.18; *p* = 0.920) did not change the conclusion.

None of the baseline-adjusted secondary T2 comparisons was statistically significant: SpO_2_, −0.89 percentage points (95% CI −1.81 to 0.03; *p* = 0.058); RR, 3.85 breaths/min (95% CI −0.69 to 8.40; *p* = 0.093); and HR, 3.17 beats/min (95% CI −6.13 to 12.48; *p* = 0.487). In the exploratory longitudinal analyses, FiO_2_ decreased over time (Greenhouse–Geisser-corrected F [1.54,37.00] = 12.77, *p* = 1.99 × 10^−4^, partial η^2^ = 0.347), but neither the overall group effect (*p* = 0.599) nor the group-by-time interaction (*p* = 0.108) was significant. Time effects were also observed for SpO_2_ (*p* = 2.25 × 10^−6^), RR (*p* = 7.73 × 10^−9^), and HR (*p* = 8.42 × 10^−7^), with no group-by-time interactions (*p* = 0.296, *p* = 0.187, and *p* = 0.352, respectively).

Exploratory between-group contrasts were nominally different at T1 for FiO_2_ (mean difference A−B 3.31 percentage points, 95% CI 0.57 to 6.05; unadjusted *p* = 0.020; Holm-adjusted *p* = 0.061) and HR (12.46 beats/min, 95% CI 1.30 to 23.62; unadjusted *p* = 0.030; Holm-adjusted *p* = 0.091). All other time-point contrasts were non-significant, and none remained significant after Holm correction. Descriptive values and the results of the ANCOVA and mixed repeated-measures ANOVA are shown in Table 2; exploratory between-group contrasts at individual time points are reported in the text.

Exploratory post-randomization outcomes also showed no statistically significant between-group differences: hospitalization was 11.7 (5.1) days, median 10 [IQR 8–14], in group A and 9.8 (3.9) days, median 9 [8–10], in group B (mean difference 1.92 days, 95% CI −1.77 to 5.61; Welch p = 0.292; Mann–Whitney sensitivity p = 0.439); respiratory support lasted 6.0 (2.4) days, median 5 [4–8], versus 4.8 (2.7) days, median 5 [3–6] (mean difference 1.15 days, 95% CI −0.94 to 3.24; Welch *p* = 0.265; Mann–Whitney sensitivity *p* = 0.301). HFNC was used in 11/13 and 12/13 participants, respectively (Fisher *p* = 1.000). No adverse events were reported.

## 4. Discussion

The principal finding of this randomized trial was that nebulized racemic epinephrine did not demonstrate superiority over saline placebo in infants with moderate-to-severe bronchiolitis requiring respiratory support. For the primary outcome, the adjusted between-group difference in T2 FiO_2_ was −0.43 percentage points (95% CI −3.43 to 2.57; *p* = 0.772). The corresponding baseline-adjusted comparisons for SpO_2_, RR, and HR were also not statistically significant. Furthermore, no statistically significant group-by-time interaction was identified for FiO_2_, SpO_2_, RR, or HR, and none of the between-group comparisons performed at T0, T1, or T2 remained significant after Holm correction. Therefore, the randomized comparisons provide no evidence that epinephrine produced a different physiological trajectory from placebo during the six-day observation period.

Significant time effects were observed for all four physiological outcomes, indicating an overall reduction in oxygen requirement, respiratory rate, and heart rate and an increase in oxygen saturation during follow-up. These findings reflect improvement in the study population as a whole rather than a treatment-specific effect. Both randomized groups received clinically indicated supportive care, and HFNC was the predominant respiratory-support modality in both groups. Consequently, the observed improvement may reflect the natural resolution of bronchiolitis, supportive management, or a combination of these factors. However, because HFNC use was not randomized and no group without respiratory support was included, the independent contribution of HFNC cannot be estimated, and the improvement should not be attributed specifically to this intervention.

Exploratory post-randomization outcomes were consistent with the primary analysis. No statistically significant between-group differences were observed in hospitalization duration, respiratory-support duration, or HFNC use. Although hospitalization and respiratory-support duration were descriptively longer in the epinephrine group, the corresponding confidence intervals included no difference, and the distribution-robust sensitivity analyses led to the same conclusion. Potentially important chance imbalances in age and exclusive breastfeeding were present at baseline; nevertheless, separate sensitivity models adjusting for these variables did not materially alter the primary FiO_2_ estimate.

These findings align with those of Abul-Ainine et al., who conducted a randomized, double-blind, placebo-controlled trial to evaluate the short-term effects of nebulized epinephrine in infants with moderately severe acute bronchiolitis. A total of 38 infants (aged 30 days to 1 year) were randomized equally into two groups (*n* = 19 per arm) to receive either 3 mg of nebulized levo-adrenaline in 3 mL solution or 3 mL of 0.9% saline placebo, delivered over 3 min via oxygen at 6 L/min. Supportive care prior to drug administration resulted in statistically significant reductions in RR by a mean of 4.3 breaths/min (*p* = 0.003) and HR by 4.6 beats/min (*p* = 0.031), with no change in SpO_2_ or RDAI scores. Following intervention, no additional statistically significant improvements were observed in any measured outcomes at 20, 40, or 60 min in either the adrenaline or placebo group. A transient, non-significant increase in HR (+6.7 beats/min at 40 min) was noted in the adrenaline group but resolved by 60 min. Overall, between-group differences in cardiorespiratory parameters remained non-significant at all time points [20].

More recent studies have questioned the routine use of nebulized epinephrine in bronchiolitis management. Yasin et al. conducted a quasi-randomized, unblinded trial to compare racemic epinephrine with 3% hypertonic saline (HS) in children between day 1 of life to 24 months of age with moderate bronchiolitis. The intervention consisted of 0.2 mL of racemic epinephrine (2.25%) diluted with 1.8 mL distilled water, administered via nebulization on admission and then every 6 h as needed, while the HS group received 2 mL of 3% saline at intervals of 1 to 4 h. The length of hospital stay ranged from 18 to 160 h in the racemic epinephrine group (mean 45 h) and 18.5 to 206 h in the HS group (mean 74.3 h). This difference was statistically significant (*p* = 0.015), indicating a shorter hospitalization with epinephrine treatment. Secondary outcomes showed that no infants in the racemic epinephrine group required supplemental oxygen, compared to 4 infants in the HS group (mean duration 30 h), and 1 racemic epinephrine patient required intravenous fluids (24 h) versus 2 HS patients (mean 15 h). No adverse effects were reported in either group [21].

Gelbart et al. conducted a pragmatic, multicenter, open-label randomized trial to evaluate whether combining systemic corticosteroids with nebulized epinephrine could reduce the duration of positive pressure support (PPS) in children with severe bronchiolitis admitted to PICU. A total of 211 children under 18 months were enrolled, with 210 included in the primary analysis (107 in the intervention group and 103 in the control group). The intervention consisted of systemic corticosteroids equivalent to 13 mg/kg prednisolone over the first 3 days, followed by 1 mg/kg daily for 3 additional days, combined with nebulized 1% epinephrine at 0.05 mL/kg diluted to 6 mL, administered every 30 min for 5 doses, then every 1–4 h for 3 days, and subsequently as needed. Children receiving corticosteroids plus epinephrine required PPS for a mean of 26.3 h compared to 39.9 h in the control group, corresponding to an adjusted ratio of 0.66 (*p* = 0.001). This represents an approximate 14-h absolute reduction in respiratory support duration. Secondary outcomes showed a shorter time to clinician-declared readiness for PICU discharge (37.2 vs. 49.8 h; *p* = 0.005), although differences in actual PICU discharge (48.9 vs. 58.8 h; *p* = 0.053) and total hospital length of stay (101 vs. 104 h; *p* = 0.607) were not statistically significant. Subgroup analyses suggested a stronger effect in RSV-positive patients, where the adjusted ratio for PPS duration was 0.49. Adverse events occurred at similar rates in both groups, with 41/107 (38%) in the intervention group and 39/103 (38%) in controls, most commonly mild sinus tachycardia [22].

## 5. Limits of the Study

This single-center trial included only 26 participants and had no formal a priori sample-size calculation; consequently, it had limited power to detect modest treatment effects, and non-significant findings should not be interpreted as proof of equivalence. The endpoint hierarchy and analysis plan were not prospectively prespecified, and the trial was not prospectively registered. The single-blind design and clinician-directed supportive care create potential performance and co-intervention bias. Chance baseline imbalances were observed for age and breastfeeding status, while respiratory-support duration differed descriptively between groups as a post-randomization exploratory outcome. The small sample size precluded reliable multivariable adjustment. Multiple outcomes and exploratory time-point contrasts also increase the risk of chance findings, although Holm adjustment was applied within each outcome. These limitations restrict precision and generalizability.

## 6. Conclusions

In this randomized, single-blind, placebo-controlled trial involving infants with moderate-to-severe bronchiolitis requiring PICU respiratory support, nebulized racemic epinephrine did not demonstrate superiority over isotonic saline placebo. The adjusted difference in day-6 FiO_2_ was small and not statistically significant, and the corresponding baseline-adjusted comparisons for SpO_2_, RR, and HR also showed no statistically significant treatment effect. Similarly, no significant group-by-time interaction was observed for any of the four physiological outcomes, indicating that their trajectories during the six-day study period did not differ significantly between the randomized groups.

FiO_2_, SpO_2_, RR, and HR improved significantly over time across the study population. However, because these improvements occurred without evidence of differential trajectories between groups, they cannot be attributed to nebulized epinephrine. They may instead reflect the expected clinical course of bronchiolitis, the effects of supportive treatment, including respiratory and nutritional support, or a combination of these factors.

Exploratory analyses similarly showed no statistically significant between-group differences in hospitalization duration, respiratory-support duration, or HFNC use. Sensitivity analyses accounting separately for the observed baseline imbalances in age and exclusive breastfeeding did not materially alter the primary FiO_2_ estimate. Nevertheless, the limited sample size, absence of an a priori power calculation, and relatively wide confidence intervals mean that these findings should not be interpreted as proof of equivalence or as excluding potentially modest treatment effects.

Within these limitations, the present findings do not support the routine addition of nebulized racemic epinephrine to supportive care for infants with moderate-to-severe bronchiolitis requiring intensive respiratory support. Clinical management should continue to prioritize evidence-based supportive measures and careful monitoring of respiratory status. Future multicenter, adequately powered, double-blind trials should use predefined outcome hierarchies and standardized respiratory-support protocols and should investigate whether treatment effects differ according to age, viral etiology, baseline severity, or other clinically relevant patient characteristics.

## Figures and Tables

**Table 1:** Baseline characteristics and exploratory post-randomization outcomes by randomized treatment group.

	Group A (n = 13)	Group B (n = 13)
**Panel A. Baseline Characteristics**		
Sex, *n* (%)	M, 8 (61.5) F, 5 (38.5)	M, 6 (46.2) F, 7 (53.8)
Caucasian ethnicity, *n* (%)	11 (84.6)	10 (76.9)
Preterm birth, *n* (%)	4 (30.8)	4 (30.8)
Exclusive breastfeeding, *n* (%)	3 (23.1)	8 (61.5)
RSV detected, *n* (%)	6 (46.2)	7 (53.8)
Age at PICU admission, mean (SD); median [IQR], days	59.9 (58.6); 37 [19–61]	113.1 (138.5); 68 [39–93]
Bronchiolitis Severity Score, mean (SD); median [IQR]	5.3 (1.7); 4 [4–7]	5.5 (2.2); 4 [4–8]
**Panel B. Exploratory post-randomization outcomes**		
Length of hospitalization, mean (SD); median [IQR], days	11.7 (5.1); 10 [8–14]	9.8 (3.9); 9 [8–10]
Duration of respiratory support, mean (SD); median [IQR], days	6.0 (2.4); 5 [4–8]	4.8 (2.7); 5 [3–6]
HFNC, *n* (%)	11 (84.6)	12 (92.3)

Values are n (%) or mean (SD); median [IQR], as indicated. Panel A contains characteristics measured before randomization; in accordance with CONSORT reporting principles, baseline significance tests are not reported. Absolute standardized differences were: male sex 0.31, Caucasian ethnicity 0.20, preterm birth 0.00, exclusive breastfeeding 0.85, RSV 0.15, age 0.50, and Bronchiolitis Severity Score 0.12. Panel B contains exploratory post-randomization outcomes; between-group analyses are reported in the text. F: female; HFNC: high-flow nasal cannula; IQR: interquartile range; M: male; n: number; PICU: pediatric intensive care unit; RSV: respiratory syncytial virus; SD: standard deviation.

**Table 2:** Descriptive outcome values, baseline-adjusted day-6 comparisons, and exploratory longitudinal effects by randomized treatment group (*n* = 13 per group).

Panel A. Descriptive Values						
Outcome	Group A: Epinephrine			Group B: Placebo		
	T0	T1	T2	T0	T1	T2
FiO_2_, %, mean (SD)	26.9 (3.8)	26.3 (4.1)	21.9 (1.9)	28.1 (6.7)	23.0 (2.3)	22.3 (4.7)
SpO_2_, %, mean (SD)	94.7 (2.6)	97.8 (1.2)	97.3 (1.0)	95.2 (2.4)	97.3 (1.8)	98.2 (1.2)
RR, breaths/min, mean (SD)	55.2 (3.1)	50.7 (4.9)	45.7 (5.6)	56.3 (7.9)	48.3 (5.2)	41.8 (5.3)
HR, beats/min, mean (SD)	150.4 (14.4)	144.4 (15.8)	130.9 (12.8)	145.5 (17.3)	131.9 (11.2)	126.5 (10.9)
**Panel B. Inferential analyses**						
**Outcome**	**Adjusted T2 difference** **A−B (95% CI)**		**ANCOVA *p***	**Group *p***	**Time *p***	**Group × time *p***
FiO_2_, %	−0.43 (−3.43 to 2.57)		0.772	0.599	1.99 × 10^−4^	0.108
SpO_2_, %	−0.89 (−1.81 to 0.03)		0.058	0.554	2.25 × 10^−6^	0.296
RR, breaths/min	3.85 (−0.69 to 8.40)		0.093	0.264	7.73 × 10^−9^	0.187
HR, beats/min	3.17 (−6.13 to 12.48)		0.487	0.091	8.42 × 10^−7^	0.352

Panel A values are mean (SD). In Panel B, adjusted T2 differences (group A minus group B) and ANCOVA *p*-values are from models adjusted for the corresponding T0 value; FiO_2_ was the primary outcome, whereas SpO_2_, RR, and HR were secondary outcomes. Group, time, and group × time *p*-values are from exploratory mixed repeated-measures ANOVA; Greenhouse–Geisser correction was applied to time and interaction tests. Group *p* evaluates the overall between-group effect across assessments, time *p* the overall change across assessments, and group × time *p* whether temporal trajectories differed. CI: confidence interval; FiO_2_: fraction of inspired oxygen; HR: heart rate; RR: respiratory rate; SD: standard deviation; SpO_2_: peripheral capillary oxygen saturation.

## Data Availability

The original contributions presented in this study are included in the article/Supplementary Materials. Further inquiries can be directed to the corresponding authors.

## Supplementary Materials

The following supporting information can be downloaded at: https://www.mdpi.com/article/10.3390/children13091254/s1, Figure S1: CONSORT flow diagram of participant enrollment, randomization, allocation, follow-up, and analysis.

## Abbreviations

The following abbreviations are used in this manuscript:

ANCOVA	Analysis of Covariance
ANOVA	Analysis of Variance
BSS	Bronchiolitis Severity Score
CI	Confidence Interval
CONSORT	Consolidated Standards of Reporting Trials
FiO_2_	Fraction of Inspired Oxygen
HFNC	High-Flow Nasal Cannula
HR	Heart Rate
HS	Hypertonic Saline
IQR	Interquartile Range
*n*	Number
PICU	Pediatric Intensive Care Unit
PPS	Positive Pressure Support
R_0_	Basic Reproduction Number
RDAI	Respiratory Distress Assessment Instrument
RR	Respiratory Rate
RSV	Respiratory Syncytial Virus
SARS-CoV-2	Severe Acute Respiratory Syndrome Coronavirus 2
SD	Standard Deviation
SpO_2_	Peripheral Capillary Oxygen Saturation
T0	Baseline assessment
T1	Day 3 assessment
T2	Day 6 assessment
